# Low-Cost Carbon Fillers to Improve Mechanical Properties and Conductivity of Epoxy Composites

**DOI:** 10.3390/polym9120642

**Published:** 2017-11-24

**Authors:** Aamer Khan, Patrizia Savi, Simone Quaranta, Massimo Rovere, Mauro Giorcelli, Alberto Tagliaferro, Carlo Rosso, Charles Q. Jia

**Affiliations:** 1 Department of Applied Science and Technology (DISAT), Politecnico di Torino, 10129 Torino, Italy; aamer.khan@polito.it (A.K.); massimo.rovere@polito.it (M.R.); mauro.giorcelli@polito.it (M.G.); alberto.tagliaferro@polito.it (A.T.); 2 Department of Electronics and Telecommunication (DET), Politecnico di Torino, 10129 Torino, Italy; 3 Faculty of Science, University of Ontario Institute of Technology (UOIT), Oshawa, ON L1H 7K4, Canada; simone.quaranta@uoit.ca; 4 Department of Mechanical and Aerospace Engineering (DIMEAS), Politecnico di Torino, 10129 Torino, Italy; carlo.rosso@polito.it; 5 Department of Chemical Engineering and Applied Chemistry, University of Toronto, Toronto, ON M5S 3E5, Canada; cq.jia@utoronto.ca

**Keywords:** dielectric material, polymer composites, carbon nanotubes, biochar

## Abstract

In recent years, low-cost carbons derived from recycled materials have been gaining attention for their potentials as filler in composites and in other applications. The electrical and mechanical properties of polymer composites can be tuned using different percentages and different kind of fillers: either low-cost (e.g., carbon black), ecofriendly (e.g., biochar), or sophisticated (e.g., carbon nanotubes). In this work, the mechanical and electrical behavior of composites with biochar and multiwall carbon nanotubes dispersed in epoxy resin are compared. Superior mechanical properties (ultimate tensile strength, strain at break) were noticed at low heat-treated biochar (concentrations 2–4 wt %). Furthermore, dielectric properties in the microwave range comparable to low carbon nanotubes loadings can be achieved by employing larger but manageable amounts of biochar (20 wt %), rending the production of composites for structural and functional application cost-effective.

## 1. Introduction

Different kinds of composites based on polymer matrix matrices are nowadays available on the market. In particular, composites based on epoxy resins are used as high-tech materials because of their excellent mechanical properties, chemical resistance, thermal stability, and low production cost [1,2,3,4,5]. Some examples of such applications are glues, adhesives, surface coatings, and electrical insulators. In recent years, there has been an increased interest in materials such as carbon nanotubes and graphene as reinforcements for polymers in order to increase the material performances [6,7,8,9,10,11,12]. However, an important aspect related to the commercialization of carbon-polymer composites is the cost reduction that can be achieved not only by cutting down the production costs but also by reducing the cost of the filler. Additionally, carbon nanotubes (CNTs) in large amounts are difficult to disperse in the matrix through conventional mechanical mixing methods. Expensive treatments such as surface functionalization with acids, plasma, or the coupling of CNTs with polymers are essential to achieve an optimum level of dispersion [13,14]. This procedure further increases the cost of the end product and undermines the industrial application potential of CNTs.

Recently, polymer scientists and technologists have been devoting their attention to the study of ecofriendly materials derived from recycling for composites production. The development of new structural and functional materials relying on ecofriendly (i.e., biochar) sources is particularly desirable in geographical areas where biomass and other biogenic wastes are abundant [15,16,17,18]. Waste materials, obtained after removing organic components (e.g., carboxylic acids, phenols, aromatic hydrocarbon, ketons, alcohools, etc.) by pyrolysis, can be used as a new type of carbon filler to increase the carbon percentage in the final products [19,20].

In this work, we investigate the mechanical and electrical (i.e., microwave) behavior of two different kinds of epoxy resin composites prepared using multiwalled carbon nanotubes (MWCNTs) and maple biochar as fillers. The impact of each filler on the composites’ mechanical and microwave properties is evaluated related to their concentration, as well as their chemical and physical properties.

## 2. Materials and Methods

Two biochar materials were used, pristine biochar (BC) and heat-treated biochar (BCHT). BC was made from maple wood by pyrolysis under an inert atmosphere, while BCHT was produced by heating BC at 950 °C under nitrogen for 4 h. Heat treatment is known to further carbonize biochar and make it more conductive. For comparison, MWCNTs were purchased from Nanothinx, Platani Rio-Patras, Greece.

The morphology of biochar materials, MWCNTs and their dispersion in the composites were studied by Field Emission Scanning Electron Microscope (FESEM-ZEISS SUPRA-40TM, Oberkochen, Germany). Composite specimens were fractured in liquid nitrogen and coated with a chromium (5 nm) layer to obtain cross-section images and to avoid charging effects, respectively.

Reactivity and specific surface area (SSA) of the two biochar materials were evaluated by thermal analysis and physio-sorption with nitrogen gas, respectively. Thermogravimetric analysis (TGA) and Differential Scanning Calorimetry (DSC) analyses were carried out with an air flow of 50 mL/min, by using a SDT Q600 analyzer (TA Instruments, Newcastle, DE, USA). Samples of approximately 30 mg were gradually heated from 30 to 1000 °C at a rate of 5 °C/min.

The specific surface area of the biochar powders was determined by N_2_ adsorption with a Nova 1200e surface analyzer (Quantachrome, Boynton Beach, FL, USA). Samples were degassed under vacuum for 3 h at 250 °C prior to measurement.

The structural quality of the biochar carbon materials and MWCNTs was investigated with Raman spectroscopy (Renishaw^®^ Ramanscope InVia H43662 model, Gloucestershire, UK) with a green laser (λ = 514 nm). Organic functional groups characterizations of biochar and biochar HT were performed through IR spectroscopy by using a Nicolet 5700 FTIR spectrometer (ThermoFisher Scientific, Waltham, MA, USA). equipped with a diamond crystal.

Epoxy resin LPL (Cores Ocean) was used as matrix to produce polymer composites with biochar materials for the electrical and mechanical characterizations. A comparison was made with composites produced from commercial multiwalled carbon nanotubes (Nanothinx NTX-4 (MW8), Platani Rio-Patras, Greece, purity > 90%).

### Composite Preparation and Analysis

Biochar pellets were grinded using an industrial pulverizer (Savatec BB90E, Torino, Italy) to an average size of 10 µm and dispersed in the epoxy resin, according to the recipe shown in Table 1. An overhead mixer (ULTRA-TURRAX T18, IKA, Wilmington, NC, USA) was used at 20,000 rpm for 2 min to obtain an optimum dispersion of the fillers in the matrix. Entrapped gas bubbles were eliminated through sonication (Elma sonic S15H, Singen, Germany) for 15 min. Afterwards, degassing was carried out in a low vacuum chamber (50 mbar) for 20 min. Finally, the composites were molded in dog bone shape (ASTM D 638-4, West Conshohocken, PA, USA) and cured in an oven at 60 °C for 4 h. Dimensional accuracy was ±0.1 mm for all samples. Five samples for each concentration were prepared to test reproducibility and allow statistical analysis of the results [21,22].

The tensile behavior of the composites prepared with MWCNTs (MW8) and biochar materials was analyzed using an MTS Q-test10 tensile testing machine (Eden Praire, MN, USA). All samples were tested with a load cell of 10 kN and a strain rate of 1 mm/min according to ASTM standard D-638. Stress vs. strain data were acquired and compared with blank epoxy resin and between samples.

Composites and pristine epoxy resin were characterized for their electrical properties in the microwave frequency range. Characterization was performed on samples of dimensions 34 mm × 34 mm × 20 mm using a commercial open-ended coaxial sensor (Agilent 85070D, Santa Clara, CA, USA) and a network analyzer (Agilent E8361A). The main advantage of this method is its smaller sample size (<20 mm in diameter) compared to the free-space method. This method is also less sensitive to sample dimension than waveguide methods. Thus, only small quantities of filler materials are needed for the measurement.

A drawback of this method is that the roughness of the surface should be smaller than a given value in order to ensure good contact with the sensor. The measurements were made considering different positions on the surface of the sample. DC (Direct Current) conductivity measurements were performed with a four-probe setup (Keithley-328 High Current Source Measure Unit coupled with a nano-amperometer, Tektronix, Beaverton, OR, USA). Five measurements on each samples were performed to ensure the reproducibility of the results.

## 3. Results and Discussion

### 3.1. Morphology of Biochar and Heat-Treated Biochar

**Figure 1:**
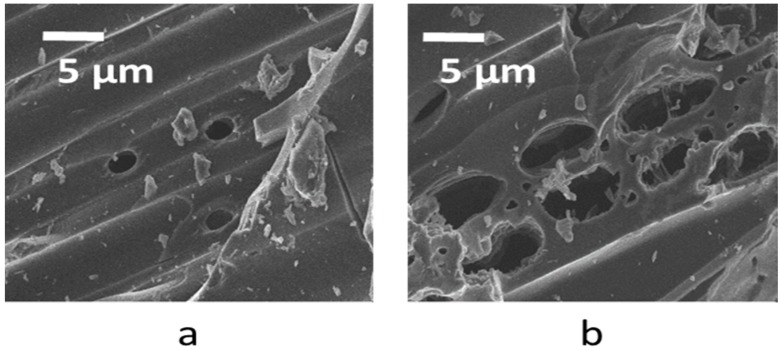
FESEM (Field Emission Scanning Microscopy) images of (**a**) biochar (BC) and (**b**) heat-treated biochar (BCHT).

Figure 1a,b respectively show the surface morphology of the biochar and heat-treated biochar samples, determined with FESEM (Field Emission Scanning Microscopy). It is anticipated that they have a similar surface morphology as they were manufactured from the same biomass precursor (maple wood). Heat treatment further carbonizes the biochar sample by reducing oxygen, hydrogen, and nitrogen contents. Consequently, heat-treated biochar should be more electrically conductive and have a surface that is more hydrophobic. The large pores in micrometers size as shown in Figure 1a,b were part of the wood structure (vessel lumen) that was retained after pyrolysis. Incidentally, the heat-treated biochar sample (BCHT) had larger vessel lumens in its precursor than the untreated biochar (BC). It should be pointed out that heat treatment can alter micro pores, which could lead to changes in SSA. However, the change in micro pores is not resolvable by images in Figure 1a,b.

### 3.2. Thermogravimetric Analysis

**Figure 2:**
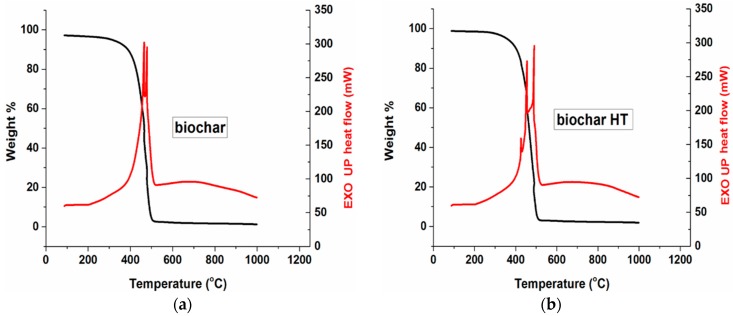
TGA-DSC curves of (**a**) biochar and (**b**) biochar HT.

Thermograms for maple biochar and HT maple biochar are reported in Figure 2a,b, respectively. Maximum combustion rate occurs at about 450 °C in both cases. Although none of the samples were subjected to acid treatment, the residual amount of ashes (stemming from the oxidation of biochar’s inorganic components: K, Ca, Na, Mg, etc.) is quite low (2.45 wt % for BC and 3.0 wt % for BCHT).

The difference between BC and BCHT in the DSC graph is likely due to their differences in chemical composition and morphology, which would determine the rate of oxidation at different temperatures. For example, as illustrated by FESEM micrographs, the macro pores diameter for the HT maple biochar have approximately a double size compared to those of its non-treated counterpart (≈5 µm vs. 2 µm, respectively). The HT maple biochar’s pores are likely more oxygen-accessible and thus combustion could begin at lower temperatures. Furthermore, the FESEM analysis revealed a relevant heterogeneity in the macro pores size of the heat-treated sample, causing the combustion process to be multi-stepped.

### 3.3. Surface Area Measurement

**Figure 3:**
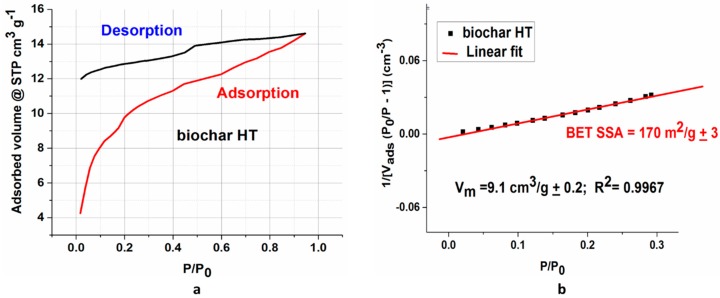
(**a**) Biochar HT surface characterization; (**b**) Complete nitrogen adsorption-desorption. BET (Brunauer–Emmett–Teller) linear fit performed on the desorption branch.

Figure 3 reports the complete N_2_ adsorption-desorption isotherm for the HT sample and the BET (Brunauer–Emmett–Teller) fit performed in the linear range of the desorption branch. HT biochar (as well as its non-heat-treated counterpart) shows a gas adsorption behavior belonging to the type II isotherms family (according to the International Union of Pure and Applied Chemistry (IUPAC) classification), which is typical of non-porous or microporous materials. Indeed, the adsorption branch shows the unrestricted formation of an adsorption monolayer (with a monolayer volume *V*_m_ of 9.1 cc/g) and then of multilayers. The H3-like type open hysteresis is consistent with the behavior of macro porous layered materials (e.g., clays), as has been already reported in literature [23,24] and in the 1985 IUPAC technical report about porous materials. The biochar layered structured is confirmed by the FESEM micrographs (Figure 1). In addition, the low pressure hysteresis without closure point is not uncommon to layered materials, especially those containing micro pores besides macro porosity [23,24].

As expected, the SSA for the HT maple biochar is two orders of magnitude higher than that of the non-treated biochar (170 ± 3 m^2^/g vs. 3.4 ± 0.3 m^2^/g). In fact, organic residues brought about by the incomplete de-methanation, de-carboxylation, and de-carbonylation of oligosaccharides remain in the biochar’s pores after the pyrolysis has plugged them. The thermal treatment carried out on the HT maple biochar unclogs the macro pores, consequently increasing the SSA [25].

### 3.4. Raman Spectroscopy

**Figure 4:**
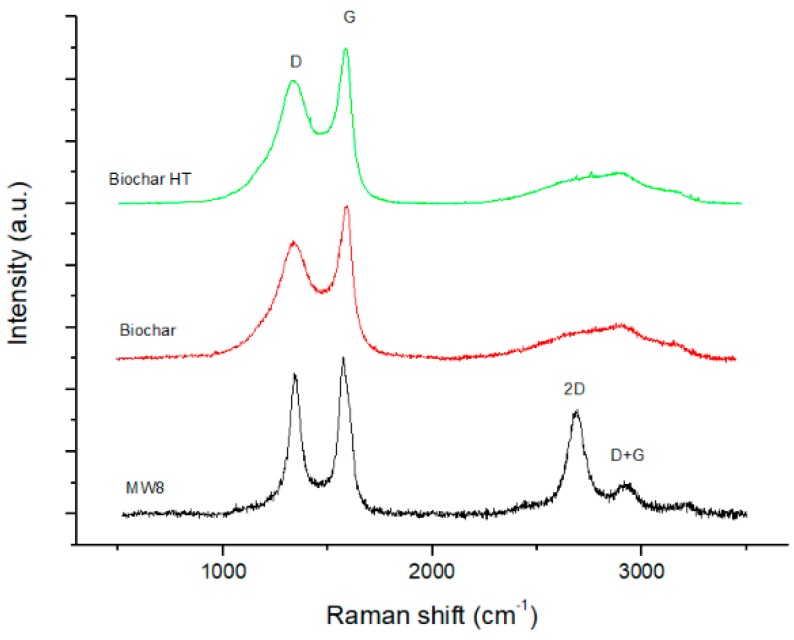
Raman spectra of MWCNTs (Multi-Walled Carbon Nano Tubes), biochar and biochar HT.

Raman spectra of commercial MWCNTs (MW8) and biochar materials, collected from 500 to 3500 cm^−1^, are illustrated in Figure 4. Raman was used to study the graphitic behavior of the three carbon species under analysis. The Raman spectrum of carbon materials is usually comprised of two relevant ranges with different features; the first set of features, located in the 1000–1700 cm^−1^ range is related to the D and G bands of carbon bonds [26]. These features are used to estimate defects (D) and graphitization grade (G), respectively. The second spectral range (2200–3500 cm^−1^) contains the second-order Raman spectrum. In this frequency range it is possible to identify the overtones of the D vibration mode, namely, the G’ or 2D second-harmonic, and the second order product of the D and G peaks.

The Raman spectra for MWCNTs show a strong second order peaks in the range 2500–3500 cm^−1^. They are called 2D and D + G peaks and are related to the sp2 graphitic structure. These peaks are less consistent in the biochar samples. This means that, as expected, the graphitic behavior is more present on MWCNTs with respect to the biochar samples. Biochar and biochar HT have similar Raman spectra (curves 2 and 3) that do not report such pronounced second order peaks.

### 3.5. Morphology of the Composites

**Figure 5:**
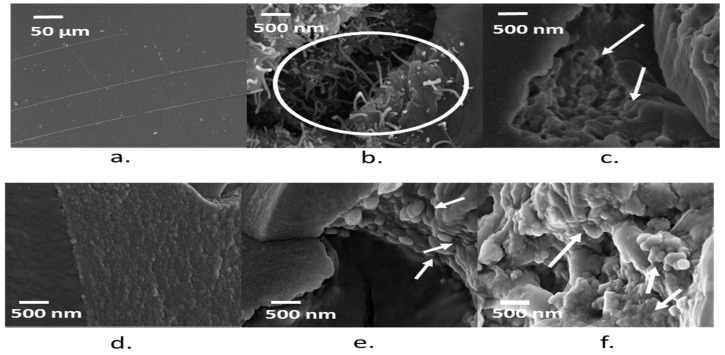
Morphology of neat epoxy (**a**) MWCNTs 1 wt %; (**b**) biochar 2 wt %; (**c**) biochar HT 2 wt %; (**d**) biochar 20 wt %; (**e**) biochar HT 20 wt %; (**f**) Composites. Arrows indicate phenomena of crack bridging and obstruction by the various fillers used.

FESEM images (Figure 5a) show that the pristine epoxy possesses a layered structure with a smooth surface and that carbon fillers are well dispersed within the polymer matrix. Biochar particles are dispersed evenly and uniformly throughout the matrix. Large agglomerates can be seen in the specimen containing higher percentages of the filler due to their high volume and surface area (Figure 5e,f). The biochar particles fix firmly in the matrix, as seen in the FESEM images. A homogeneous dispersion of the filler particles is instrumental in improving composite properties compared to the neat polymer. In fact, biochar sub micrometric structures evenly distributed across the epoxy resin matrix hinder the crack propagation caused by the accumulation of micro-cracks. During the application of the tensile load, these particles are de-bonded/pulled out of the matrix, as shown in the FESEM images, and thus help the matrix to withstand higher loads. Conversely, the MWCNTs are dispersed in large chunks and bridge between the layers of the matrix. Such a geometry promotes matrix interlocking, leading to enhanced tensile properties.

### 3.6. Mechanical Analysis

**Figure 6:**
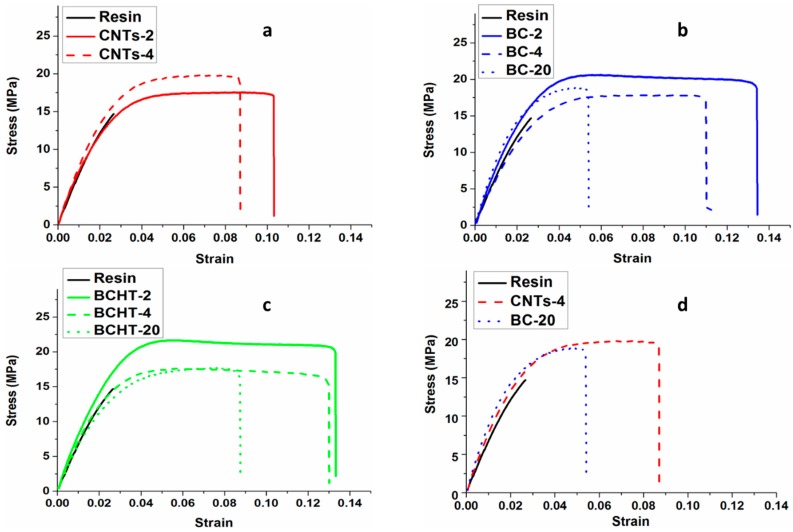
Stress vs. strain behavior of blank epoxy compared to (**a**) MWCNTs 2 and 4 wt %; (**b**) biochar 2, 4 and 20 wt %; (**c**) biochar HT 2, 4 and 20 wt %; (**d**) Comparison between blank epoxy, biochar 20 wt %, and MWCNTs 4 wt %.

The mechanical behavior of the polymer matrix was modified upon the addition of the three different carbon fillers. The stress-strain behavior of the various samples is shown in Figure 6. From the curves, the information on ultimate tensile strength, stiffness (Young Modulus), strain at break, and tensile toughness of the different types of composites are obtained. It can be seen that the brittle behavior of the epoxy resin is turned into a ductile beahvior after the addition of small amounts of any of the carbon fillers. Furthermore, the filler’s type and concentration were found to affect the mechanical behavior of the composite. Although a detailed analysis of the values of the various parameters will be discussed later and the addition of any amount of any carbon fillers led to improved mechanical properties, it is evident from Figure 6 that the larger improvement is obtained by adding 2 wt % of either heat-treated or non-heat-treated biochar. Higher biochar wt % showed reduced ultimate strength and elongation i.e., a trend towards semi-brittle behavior. This behavior could be attributed to the high specific volume of the filler (20 wt %). Oisik Das et al., 2016 [27] also reported the improved mechanical properties of the biochar polypropylene composites and the same semi-brittle trend at high biochar wt %. The increase in the mechanical strength of the composite can be ascribed to the biochar particles’ ability to obstruct cracks onset and accumulation, as shown in the FESEM images. Cavitation/de-bonding of the filler from the matrix due to applied stress is also evident from the FESEM analysis. Cavitation and de-bonding effects are usually responsible for enhancing composites’ mechanical properties [28,29]. The cross-link ratio of the epoxy resin may also have improved due the addition of biochar fillers, which effectively block the molecular motion in the polymer matrix, reducing its deformability and thus strengthening the polymer matrix [30,31]. The increase in the thermal conductivity of the composite due to the higher thermal conductivity of filler can also play a role, as it leads to a lower concentration (or faster diffusion) of heat generated by plastic deformation in a given section of the composite and hence to the ability to withstand a larger strain before locally reaching glass transition temperature [32].

**Figure 7:**
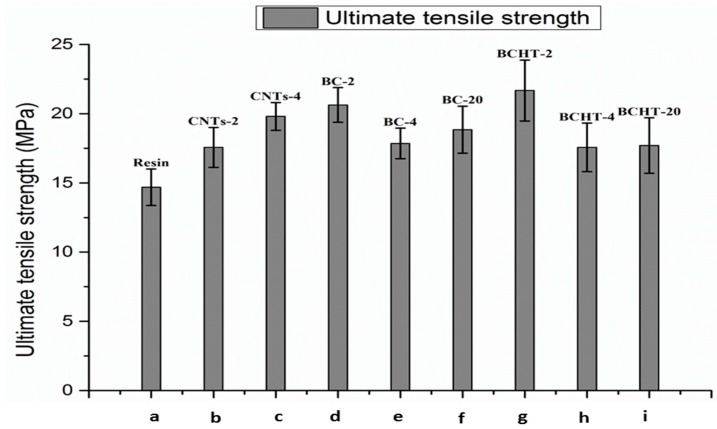
Ultimate tensile strength comparison. (**a**) Neat epoxy; (**b**) MWCNTs 2 wt %; (**c**) MWCNTs 4 wt %; (**d**) biochar 2 wt %; (**e**) biochar 4 wt %; (**f**) biochar 20 wt %; (**g**) biochar HT 2 wt %; (**h**) biochar HT 4 wt %; (**i**) biochar HT 20 wt %.

The addition of the various carbon fillers enhanced the tensile strength of the neat epoxy matrix. Higher loading of the MWCNTs (4 wt %) enhanced the UTS (Ultimate tensile strength), while an opposite trend can be seen in the biochar and heat-treated biochar filler. The composite mechanical properties first increase with the addition of the filler particles, and then start to deteriorate upon higher loading of the filler i.e., 20 wt %. Spanoudakis et al. [33] reported the similar behavior of the epoxy matrix by the addition of micrometric-size silica particles. Nan et al. [34] also confirmed the similar behavior of the biochar/PVA (Polyvinyl alcohol)matrix polymer composites. A 48% increase of in the maximum load-bearing value (i.e., ultimate tensile strength) compared to the pristine epoxy resin is observed for the 2 wt % HT biochar sample (see Figure 7).

**Figure 8:**
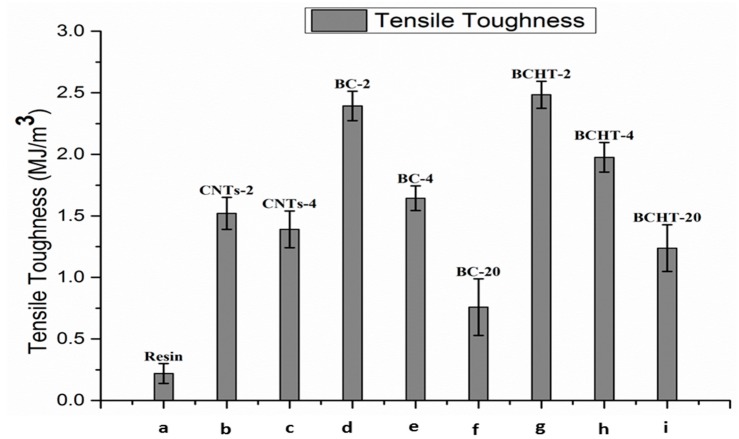
Tensile toughness comparison: (**a**) Neat epoxy; (**b**) MWCNTs 2 wt %; (**c**) MWCNTs 4 wt %; (**d**) biochar 2 wt %; (**e**) biochar 4 wt %; (**f**) biochar 20 wt %; (**g**) biochar HT 2 wt %; (**h**) biochar HT 4 wt %; (**i**) biochar HT 20 wt %.

The enhanced mechanical properties of the matrix can be explained in terms of stress transfer from the matrix to the biochar filler due to its high surface area. In fact, when equal wt % of biochar are taken in to account, the HT biochar shows superior mechanical properties compared to the non-treated samples because of its large pore size, as shown in the FESEM images. Larger pores enhance the wettability with the matrix and, consequently, the composite of heat-treated biochar performed better than its counterparts. The addition of a small amount of the filler enhanced the overall load-bearing capacity/tensile toughness of the matrix. An increase of 10 times in the tensile toughness was achieved using 2 wt % of the heat-treated biochar, as shown in Figure 8. Biochar-based fillers performed better than MWCNTs at the same wt %. The load-bearing capacity deteriorated with the higher loading of the filler. The trend remained same in all types of the carbon fillers used. The lower tensile toughness at higher loading of biochar fillers was due the change of the plastic behavior into semi-brittle behavior [35]. At higher filler loading, the cross-linking and stacking of the polymer system increases and thus reduces the movement of polymer molecules. Consequently, the matrix shows a brittle behavior [36]. The change in the mechanical properties of the composites at higher concentrations of biochar fillers is probably due to uneven dispersion of the particles in the low density polymer matrix and the powder-like nature of the biochar filler [37].

### 3.7. Complex Permittivity Measurements

**Figure 9:**
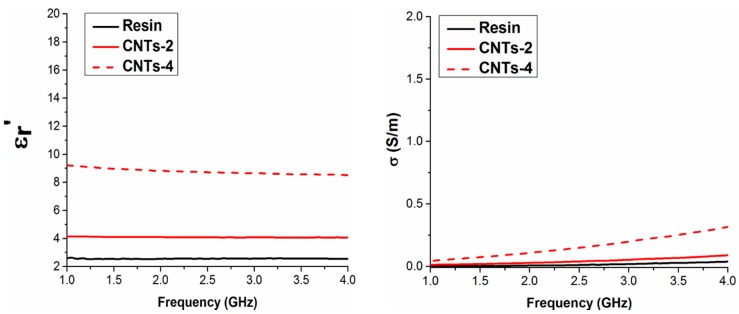
Real part of permittivity (**left panel**) and conductivity (**right panel**) in the microwave range for pure epoxy and epoxy composites filled with 2 and 4 wt % of MWCNTs.

**Figure 10:**
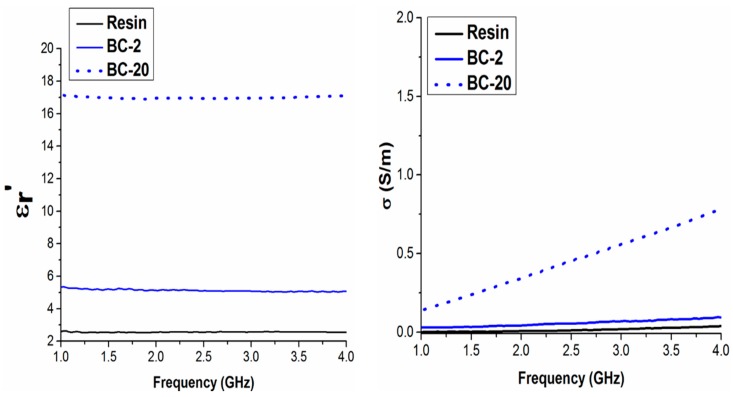
Real part of permittivity (**left panel**) and conductivity (**right panel**) in the microwave range for pure epoxy and epoxy composites filled with 2 and 20 wt % of biochar.

The real part of permittivity and the conductivity in the microwave range (1–4 GHz) of the pure resin and the resin filled with MWCNTs 2 and 4 wt % vs. frequency is shown in Figure 9. As expected, the real part of the dielectric constant and the conductivity values both increase as filler percentage is increased. In Figure 10 the results obtained for biochar with 2 and 20 wt % are shown. In order to achieve a significant increase of the values of the complex permittivity, it is necessary to load the composite with at least 10 wt % of biochar. Conversely, MWCNTs bring about a dielectric constant increase (i.e., real part of complex permittivity), even at lower concentrations, because of their low percolation threshold required to modify a composite’s electrical properties [38,39,40,41,42]. Moreover, MWCNTs-containing composites also benefit from the carbon nanotubes’ high aspect ratio (800~1000), resulting in an improved electrical conductivity in the microwave range (compared to the bare resin). On the other hand, biochar’s three-dimensional structure results in worse particle interconnectivity, consequently raising the percolation threshold to create a conductive path throughout the composite. In fact, DC conductivity measurements carried out on biochar samples showed an insulating behavior. On the other hand, conductivity values for pure biochar and biochar HT are of the order of 0.1–0.2 S/m.

**Figure 11:**
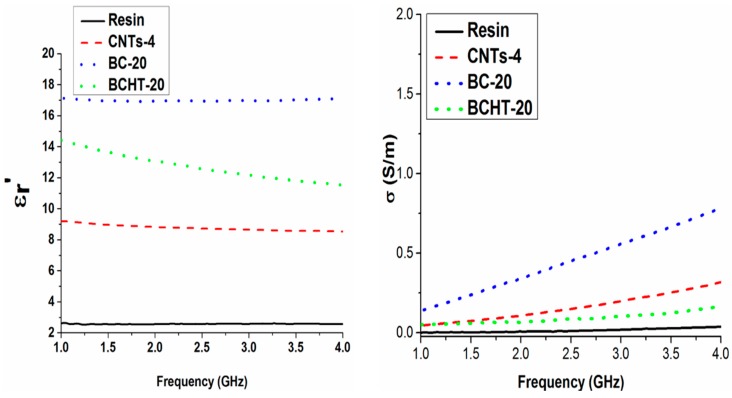
Comparison among the real part of permittivity (**left panel**) and conductivity (**right panel**) in the microwave range for pure epoxy and epoxy composites filled with 4 wt % of MWCNTs and 20 wt % of each biochar type.

**Figure 12:**
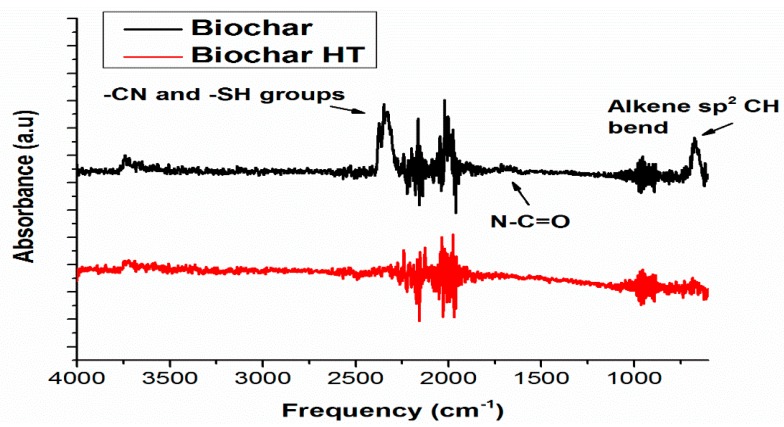
IR spectra of biochar and biochar HT.

It is worth noting the biochar real permittivity trend against the frequency of the applied field. A decreasing trend, due to the relaxation of polar organic groups (e.g., OH, C=O, COOH, etc.), is observed in the non-heat-treated sample. Conversely, the lower electronic delocalization due the removal of alkene, amide, nitrile, moieties is responsible for the lower electrical conductivity at the microwave frequencies shown by the HT samples. Furthermore, the removal of thiols by heat treatment contributes to the electrical conductivity’s reduction (see Figure 11). Indeed, sulphur electron lone pairs play a role in the conductivity at the microwave frequencies. Such assumptions are supported by the IR spectra of biochar and biochar HT (see Figure 12), where it is possible to observe the decreasing of functional groups after thermal treatment.

Finally, in Figure 11 the samples of biochar 20 wt %, non-heat-treated and activated, are compared with MWCNT 4 wt %. The results show that similar microwave properties (permittivity and electrical conductivity in the microwaves) of composites can be obtained by biochar and MWCNTs, but at a higher wt % in the biochar case. Last but not least, the different graphitic structures of MWCNTs with respect to biochar, as reported by Raman analysis, could play a role in the electrical behavior.

## 4. Conclusions

The mechanical and electrical performances of two types of carbon filler (MWCNTs and biochar) dispersed in epoxy resin were investigated. These two types of filler were compared because MWCNTs requires somewhat harsh chemical processing for production, whereas biochar stems from a renewable low-cost source.

From the point of view of mechanical properties, the most remarkable improvement (in particular in ultimate tensile stress and elongation at break) was obtained for a 2 wt % load of biochar. Still, at 20 wt %, the biochar filler over performed MWCNTs composites in strength and stiffness, but showed somewhat inferior properties in the plastic zone (see Figure 6).

From the point of view of microwave properties, on the other hand, the properties obtained by using 4 wt % of MWCNTs were improved by using 20 wt % of maple wood biochar.

It has to be noted that 4 wt % is about the maximum dispersion limit of MWCNTs, and uniform dispersion can be obtained only with dedicated additional steps compared to that required for the standard filler dispersion. Conversely, no difficulties are incurred in by dispersing as much as 20 wt % of biochar in resin. In addition, biochar is a low-cost, ecofriendly, renewable material.

The results reported in this paper suggest that further investigation of the impact of parameters such as filler particle size, shape, and orientation in the matrix are warranted in order to enhance the overall mechanical performances of biochar-based polymer composites.

## Figures and Tables

**Table 1:** Recipe for composite preparation.

S/No.	Sample ID	Resin (g)	Hardener (g)	Filler (g)
1	Blank epoxy	66.67	33.33	0
2	2%	65.33	32.67	2
3	4%	64.00	32.00	4
4	20%	53.33	26.67	20
